# Sorting Nexin 5 Plays an Important Role in Promoting Ferroptosis in Parkinson's Disease

**DOI:** 10.1155/2022/5463134

**Published:** 2022-05-05

**Authors:** Zifeng Huang, Jiajun Han, Peipei Wu, Chunxiao Wu, Yaohua Fan, Lijun Zhao, Xiaoqian Hao, Dongfeng Chen, Meiling Zhu

**Affiliations:** ^1^Traditional Chinese Medicine Innovation Research Center, Shenzhen Hospital of Integrated Traditional Chinese and Western Medicine, Guangzhou University of Chinese Medicine, Shenzhen 518104, China; ^2^Department of Anatomy, The Research Center of Basic Integrative Medicine, Guangzhou University of Chinese Medicine, Guangzhou, China

## Abstract

Parkinson's disease (PD) is a common neurodegenerative disease in the elderly, which is related to brain iron metabolism disorders. Ferroptosis is a newly discovered iron-dependent programmed cell death mode, which has been considered an essential mechanism of PD pathogenesis in recent years. However, its underlying mechanisms have not been fully understood. In the present study, the PD rat model and PD cell model were induced by 6-hydroyxdopamine (6-OHDA). The results showed that the expression of Sorting Nexin 5 (SNX5) and the level of ferroptosis will increase after treatment with 6-OHDA. Consistent with these results, ferroptosis inducer erastin synergistically reduced the expression of glutathione peroxidase 4 (GPX4) and increased the expression of SNX5 in the PD cell model, while ferroptosis inhibitor ferrostatin-1 (Fer-1) inhibited the decrease of GPX4 and the increase of SNX5 in the PD cell model. Knockdown of SNX5 in PC-12 cells could reduce intracellular lipid peroxidation and accumulation of Fe^2+^ and significantly inhibit the occurrence of ferroptosis. In conclusion, the present study suggested that SNX5 promotes ferroptosis in the PD model, thus providing new insights and potential for research on the pharmacological targets of PD.

## 1. Introduction

Parkinson's disease (PD) is the second-largest neurodegenerative disease affecting the global population [1]. From 2005 to 2015, the global death toll from PD increased by about 40%, and the incidence of PD increased faster than other nervous system diseases. In our country, the incidence of PD is 17% over the age of 65 [2]. PD is characterized by a decrease in the number of dopaminergic neurons and an abnormal accumulation of *α*-synuclein [3, 4]. The decrease of dopamine will lead to tremor, bradykinesia, rigidity, posture balance disorder, and other clinical motor symptoms [5, 6]. However, no cure has been found for PD. Therefore, there is an urgent need to develop new targets to improve the diagnosis and treatment of PD patients.

Iron ions are essential for normal brain development and cognitive function, and iron-dependent enzymes and ferritin are necessary for synapses, myelin formation, and neurotransmitter production and transformation [7, 8]. However, iron is an oxidant, and excessive deposition can also cause severe damage to the cell. Excessive free iron causes oxidative nitrification stress, inflammation, and excitatory toxicity, resulting in cell damage and neurodegeneration [9]. The consequences of metabolic disorders of iron ions may be the main cause of PD. Relevant studies have shown that ferroptosis participates in the pathogenesis of PD. Iron overload and lipid peroxidation are the main characteristics of ferroptosis [10]. Ferroptosis is related to glutathione peroxidase 4 (GPX4) activity [11–13]. However, the mechanism of ferroptosis in PD remains unclear.

In the previous work, the difference in the expression of Sorting Nexin 5 (SNX5) between the PD rat model and the normal rat was determined by iTRAQ protein sequencing analysis [14]. SNX5 is one of the most important proteins in the endosome sorting component retromer, which is mainly expressed in the endosome and is involved in the identification, transport, and unloading of substances between various organelles in the cell [15, 16]. The content of SNX5 in cells affects the endocytosis sorting and other component transportation of cells [16, 17]. It has been reported that the SNX family regulates protein homeostasis in nerve cells, and incorrect protein transport is related to the development of many neurodegenerative diseases, including PD [18]. However, the relationship between SNX5 and ferroptosis in PD has not been explored and deserves further study.

In this study, we investigated the specific mechanism of SNX5 in the appearance of PD. The results confirmed that the knockdown expression of SNX5 in the PD cell model could reduce the level of lipid peroxidation and inhibit the occurrence of ferroptosis. Therefore, SNX5 had the effect of promoting ferroptosis in PD, thus providing new insights and potential for research on the pharmacological targets of PD.

## 2. Materials and Methods

### 2.1. Animal

A total of 36 male Sprague-Dawley (SD; 180 g-200 g; 6-8 weeks of age) rats were purchased from Guangdong Zhiyuan Biomedical Technology Co. LTD., and animal certificates were provided (no. 110322210101368063). All animal experiments are permitted by the Animal Ethics Committee of Guangzhou University of Chinese Medicine (no. 20220330002). And all study conducted following the guidelines for the care and use of experimental animals of the National Institutes of the health of the United States and provided free access to food and water. The rats were divided into three groups (control group, sham group, and model group), with 12 rats in each group.

### 2.2. PD Rat Model Establish

The PD rat model was established by injecting 6-hydroyx dopamine (6-OHDA) (H116-5MG, Sigma-Aldrich, MO, USA) into the one-sided medial forebrain bundle (MFB) target of the rat [19]. Dissolve 6-OHDA powder in 0.2% ascorbic acid to prepare 8 mg/ml 6-OHDA solution. Rats were anesthetized by intraperitoneal injection of 30 mg/kg of 3% sodium pentobarbital. After rats were anesthetized, their heads were shaved and fixed on the brain stereotaxic frame. Then, the rat head skin was sterilized with 75% alcohol, and the rat head skin was cut with scissors to find the location of the anterior fontanel, which was then used as the origin for positioning. The rats of the model group were injected with 25 *μ*g 6-OHDA at MFB (A/P = 4.4, M/L = 1.2, and D/V = 7.8 mm). When using the cranial drill, avoid damaging the brain of rats due to excessive force, and the microsyringe was used to slowly insert to the brain. Before awakening, rats should use a thermal insulation pad to keep the rats' body temperature at 36.5°C. In order to prevent infection, penicillin (100,000 units) was injected intraperitoneally after the operation. The experimental operation in the sham group was exactly the same as that in the model group, except that 6-OHDA was replaced with an equal volume of saline solution. The rats in the control group were raised normally without any treatment.

### 2.3. Behavioral Test

The spontaneous rotation behavioral test was performed 28 days after the establishment of the PD rat model. After intraperitoneal injection of apomorphine (APO, 017-18321, Wako Pure Chemical Industries, Osaka, Japan) at 0.5 mg/kg, the rats were placed on a circular platform with a diameter of 50 cm. Rotations were manually counted 10 minutes after the APO injection. Record the number of left-hand rotations for 30 minutes. The model rats with a rotational speed over 7 turns/minutes were considered suitable for further analysis.

### 2.4. Immunofluorescence

Brain perfusion was performed on three rats in each group, followed by paraffin embedding and sectioning. Firstly, paraffin wax dewaxing with xylene for 15 minutes and hydrated with ethanol (anhydrous ethanol, 95% ethanol, 90% ethanol, and 70% ethanol for 10 minutes each). Secondly, antigen repair by completely immersing the tissue slides in 0.01 M citric acid buffer, boiling for 6 minutes, and then cooling to room temperature, repeating for 2 times. Then, sections were washed 1 time in PBS and permeabilized with an immunostaining permeabilization buffer with saponin (P0095-100 ml, Beyotime, Shanghai, China) for 10 minutes and were blocked with 10% goat serum (C0265, Beyotime, Shanghai, China) for 20 minutes at room temperature. After blocking, the sections were incubated overnight with tyrosine hydroxylase (TH, 1 : 2000, 25859-1-AP, Proteintech, Wuhan, China). On the next day, sections were washed in PBS and incubated with secondary antibody (1 : 1000, ab150077, Abcam, MO, USA) for 1.5 h at 37°C and washed in PBS. The nucleus was stained with DAPI (1 : 1000, C1002, Beyotime, Shanghai, China) for 5 minutes at room temperature and then washed with PBS. At last, the sections were sealed with antifade mounting medium (P0126-5 ml, Beyotime, Shanghai, China) and imaged by Cytation 5™ Cell Imaging Multi-Mode Reader (BioTek, VT, USA).

### 2.5. Cell Culture and Reagents

PC-12 cells were obtained from the Chinese Academy of Sciences. Cells were cultured in RPMI 1640 medium (C11875500BT, Gibco, CA, USA) containing 10% fetal bovine serum (XC6936T, Guangzhou, China) and 1% Penicillin-Streptomycin (15140122, Gibco, CA, USA). Erastin (HY-15763, NJ, USA) and ferrostatin-1 (fer-1, HY-100579, NJ, USA) were purchased from MedChem Express.

### 2.6. Cell Viability Assay

The MTS assay kit (G3580, Promega, WI, USA) was used to determine cell viability. PC-12 cells (1 × 10^4^/well) were plated in flat-bottomed 96-well plates. Discarding the cell culture medium after 24 hours of 6-OHDA stimulation, MTS was added to each well and incubated for 2 hours at room temperature. A microplate reader was used to record the OD values at a wavelength of 490 nm (BioTek, VT, USA). Live-death cell staining was detected using the calcein-AM/PI test kit (R37601, Thermo, CA, USA). PC-12 cells (1 × 10^4^/well) were plated in black-walled porous 96-well plates and then treated with gradient concentrations of 6-OHDA for 24 hours. Then, cells were washed before being stained for 20 minutes with 5 M PI and 5 M calcein-AM and imaged by Cytation 5™ Cell Imaging Multi-Mode Reader.

### 2.7. Western Blot Analysis

The cells were lysed in RIPA buffer (89900, Thermo, CA, USA) and centrifuged at 12,000 rpm for 15 minutes at 4°C to obtain the supernatant. The pierce BCA protein assay kit (23227, Thermo Scientific, CA, USA) was used to determine the protein content according to the manufacturer's protocol. The total protein (30 *μ*g) of the sample was separated using 10% SDS-PAGE and transferred to polyvinylidene difluoride membranes. The membrane was blocked for 1 hour at room temperature with 5% skimmed milk powder before overnight incubation with primary antibodies such as SNX5 (1 : 1000, 17918-1-AP, Proteintech, Wuhan, China), FTH1 (1 : 1000, 4393S, Cell Signaling Technology, MA, USA), GPX4 (1 : 1000,52455S, Cell Signaling Technology, MA, USA), HRP-conjugated *β*-actin (1 : 2000, HRP-60008, Proteintech, Wuhan, China), and TH (1 : 2000, 25859-1-AP, Proteintech, Wuhan, China). Rinse three times with TBST and incubate with HRP-labelled secondary antibody (1 : 1000, ab6721, Abcam, MO, USA) for one hour at room temperature. Immunoblotting Western HRP substrate (WBKLS0500, Millipore, MA, USA) was used to visualize the bands. Image J software was used to assess the density of the bands. All of the data comes from at least three different experiments.

### 2.8. Cell Transfection

GenePharma discovered and produced siRNAs that target SNX5 (m-Pack1999, RIBOBIO, Guangzhou, China). The sequence of siRNA was as follows: siRNA#1, 5′GGATGACTTCTTTGAGCAA 3 ′; siRNA#2, 5 ′ GCACAAAGGCCCTAATTGA3 ′; siRNA#3, 5′ GGAAGAGAGTGGCAGCATT3′. Lipofectamine 3000 (Lipi3000, L3000015, Invitrogen, CA, USA) was used to transfect the siRNA to the cells according to the manufacturer's protocol. Lipo3000 and siRNA were diluted for in OptiMEM (31985070, Gibco, CA, USA) and mixed gently; then, the mixture was added to the cell cultures.

### 2.9. Determination of Reduced Glutathione (GSH)/Oxidized Glutathione (GSSG) Activity

In order to detect the ratio of GSH/GSSG in the cell and animal, the GSH/GSSG Assay Kit (S0053, Beyotime, Shanghai, China) was performed according to the manufacturer's protocol. PC-12 cells (2.5 × 10^6^/well) were cultured in a 6 cm culture dish, and then, the culture media was withdrawn after a 24-hour treatment with 40 *μ*M 6-OHDA. Three rats in each group were anesthetized, and substantia nigra (SN) was collected in the unconscious state of the rats.

### 2.10. Determination of Malondialdehyde (MDA)

In order to detect the concentration of MDA in the cell and animal, the Lipid Peroxidation MDA Assay Kit (S0131S, Beyotime, Shanghai, China) was performed according to the manufacturer's protocol. Cells and tissues were lysed by IP-lysis (87787, Thermo Scientific, CA, USA) for 20 minutes at 4°C. After sample preparation, protein concentration can be measured with the pierce BCA protein assay kit to facilitate subsequent calculation of MDA content in tissues or cells per unit protein weight.

### 2.11. Intracellular Ferrous Ion Imaging

The FeRhonox-1 staining kit (SCT030, Goryo Chemical, Sapporo, Japan) was used to determine the distribution of Fe^2+^ in the cell according to the manufacturer's protocol. PC-12 cells (1 × 10^4^/well) were cultured in the black-walled porous 96-well plates overnight. The cell culture medium was withdrawn after the cells had been treated with 6-OHDA for 24 hours, and the cells were washed two times with PBS. Then, the cell media was supplemented with 1 *μ*M FeRhoNox-1 and imaged by Cytation 5™ Cell Imaging Multi-Mode Reader.

### 2.12. Statistical Analysis

The experimental data are presented as the mean ± SEM. The figures were produced by GraphPad Prism 8.0 (GraphPad Software, San Diego, CA). The comparison between multiple groups was analyzed by One-way analysis of variance followed by SNK multiple comparisons test as the post hoc test. *p* < 0.05 was considered to indicate a statistically significant difference.

## 3. Results

### 3.1. The Expression of TH Significantly Decreased in the PD Rat Model

**Figure 1:**
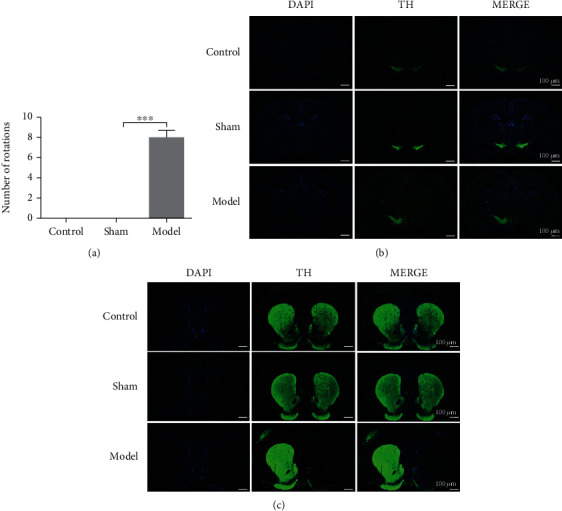
The expression of TH significantly decreased in the PD rat model. (a) The changes in the rotation number of rats after injection of APO. The changes of the control, sham, and model groups were compared. (b) Immunofluorescence was performed on the SN of the rat in the control, sham, and model groups. (c) Immunofluorescence was performed on the striatum of the rat in the control, sham, and model groups. Data were expressed as mean ± S.D.; *n* ≥ 3 per group for all the studies (^∗∗∗^*p* < 0.05).

After 28 days of modeling, the rats were intraperitoneal injection of APO to observe the number of laps to determine whether the modeling was successful. Twelve rats turned >7 times/min and reached the modeling standard (Figure 1(a)). TH is the signature protein of dopaminergic neurons in the nervous system. The IF result showed that the expression of TH in the SN (Figure 1(b)) and the striatum (Figure 1(c)) was significantly decreased in the 6-OHDA-treated hemisphere compared with the contralateral hemisphere in the model group. While the expression of TH in the control group and the sham group was symmetric.

### 3.2. The Level of Ferroptosis and the Expression of SNX5 Were Increased in the PD Rat Model

**Figure 2:**
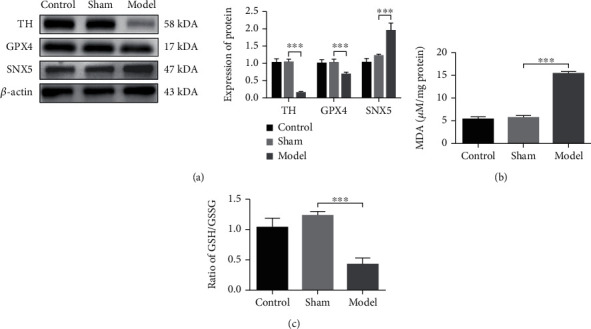
The level of Ferroptosis and the expression of SNX5 were increased in the PD rat model. (a) Western blot analysis of the expression of TH, GPX4, and SNX5. (b) The concentration of GSH/GSSG in SN of the PD rats was detected by MDA Assay. (c) The ratio of GSH/GSSG in SN of the PD rats was detected by GSH/GSSG-GLO Assay. Data were expressed as mean ± S.D.; *n* ≥ 3 per group for all the studies (^∗∗∗^*p* < 0.05).

A growing number of studies have demonstrated that ferroptosis is an essential mode of dopamine neuron death. To determine whether 6-OHDA can induce ferroptosis in the PD rat model, we examined the expression of GPX4, the concentration of MDA, and the ratio of GSH/GSSG. As shown in Figure 2(a), the expression of TH and GPX4 was decreased significantly in the model group. The concentration of MDA increased significantly in the model group, which represents the level of lipid peroxides was increased in the model group (Figure 2(b)). In addition, glutathione depletion and the decrease of GPX4 activity can lead to increased lipid peroxidation. Therefore, the ratio of GSH/GSSG decreases significantly in the model group (Figure 2(c)). The expression of SNX5 was significantly increased in the model group (Figure 2(a)), which indicated that an abnormal increase of SNX5 induced by 6-OHDA contributes to the formation of PD pathology.

### 3.3. The Expression of SNX5 and the Level of Ferroptosis Were Increased in the PD Cell Model

**Figure 3:**
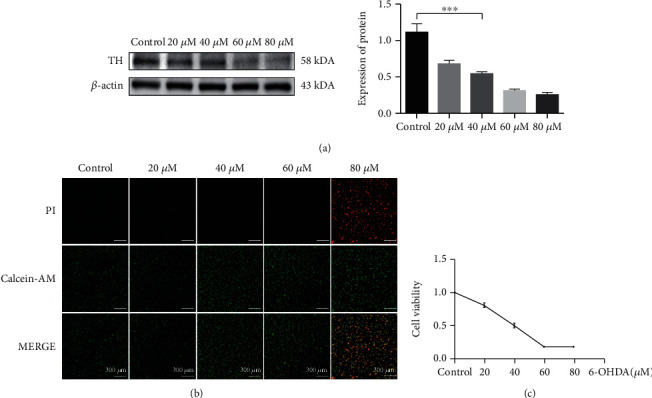
Screening the optimal stimulation concentration of the 6-OHDA to establish the PD cell model. (a) Stimulation of PC-12 cells with 20, 40, 60, and 80 *μ*M concentration of 6-OHDA for 24 hours. The expression of TH was detected by Western blotting. (b) PC-12 cells were treated with 20, 40, 60, 80 *μ*M concentration of 6-OHDA for 24 hours, and live and dead cells were detected by immunofluorescence, live cells by red PI, and dead cells by green calcein-AM. Observation of cellular changes with fluorescence microscopy. (c) PC-12 cells were treated with 20, 40, 60, 80 *μ*M concentration of 6-OHDA for 24 hours, cell viability was evaluated by the MTS assay (^∗∗∗^*p* < 0.05).

**Figure 4:**
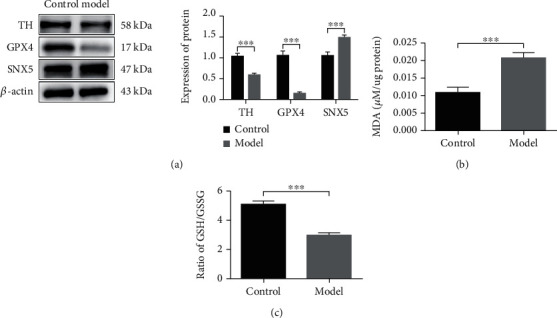
The expression of TH and GPX4 was decreased, while the expression of SNX5 was increased in the PD cell model. (a) PC-12 cells were untreated or treated with 40 *μ*M 6-OHDA for 24 h. The expression of TH, GPX4, and SNX5 was detected by Western blotting. (b) MDA Assay was used to detect the concentration MDA in PC-12 cells was induced by 6-OHDA. (c) GSH/GSSG ratio in vitro PD was detected by GSH/GSSG Assay. Data were expressed as mean ± S.D.; three independent replicates of the experiment (^∗∗∗^*p* < 0.05).

The result of MTS has the similarly trend as the expression of TH (Figure 3(a)) and live-death cell staining (Figure 3(b)), 6-OHDA shows significant cytotoxicity at high concentrations, and the IC50 value was 40 *μ*M (Figure 3(c)). Based on the above experimental results, we chose 40uM 6-OHDA as the optimal stimulation concentration for the next experiment. Our results also showed that the expression of TH and GPX4 was significantly decrease in the model group, while the expression of SNX5 was significantly increase (Figure 4(a)). To explore the level of ferroptosis in the model group, we examined the level of lipid peroxidation and the ratio of GSH/GSSG. The results showed that the concentration of MDA increased significantly (Figure 4(b)), while the ratio of GSH/GSSG decreased significantly in the model group (Figure 4(c)), which was consistent with the results in the PD rat model and suggested that 6-OHDA could induce ferroptosis. In summary, the experimental results demonstrated that SNX5 expression was upregulated in the model group compared to the control group, suggesting that SNX5 is involved in developing PD pathogenesis.

### 3.4. The Effects of Erastin and Fer-1 on PD Cell Model

**Figure 5:**
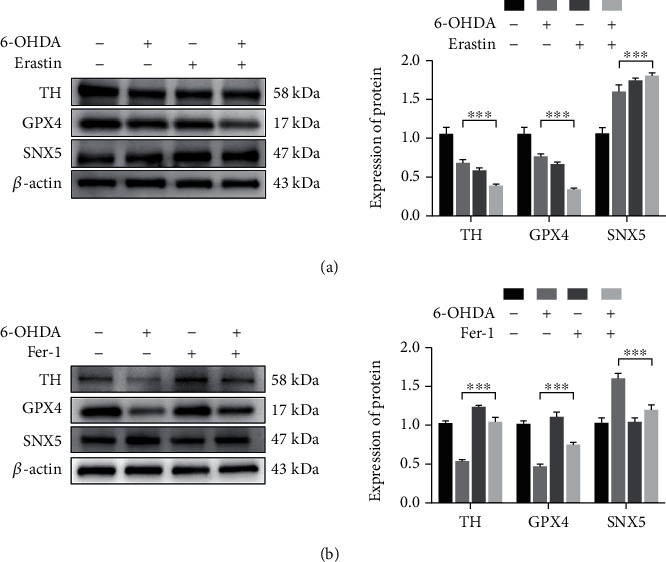
The effects of erastin and fer-1 on PD cell model. PC-12 cells were induced with 40 *μ*M 6-OHDA in combination with an inducer (1 *μ*M erastin) or inhibitor (2.5 *μ*M fer-1) of ferroptosis for 24 h. The expression of GPX4, TH, and SNX5 was detected by Western blotting (a) and (b). Data were expressed as mean ± S.D.; three independent replicates of the experiment (^∗∗∗^*p* < 0.05).

The above experiments showed that 6-OHDA induced the onset of ferroptosis in PC-12 cells and changes in the corresponding indexes. In this study, the effects of erastin and fer-1 on the PD cell model were explored. The results showed that the ferroptosis inducer erastin enhanced the role of 6-OHDA in the cells. The expression of TH and GPX4 was decreased more, and the expression of SNX5 was increased more in cells treated with the combination of erastin and 6-OHDA than in cells treated with only 6-OHDA (Figure 5(a)). However, all changes in indicators due to ferroptosis are redeemed by the ferroptosis inhibitor fer-1. Compared with the cells treated with 6-OHDA alone, the expression of TH and GPX4 did not decrease, and the expression of SNX5 did not increase in the cells treated with the combination of 6-OHDA and fer-1, indicating that the level of ferroptosis in the PD cells was inhibited by fer-1 (Figure 5(b)). In conclusion, these results further demonstrate that 6-OHDA can induce ferroptosis in PC-12 cells, and the expression of SNX5 will be upregulated when cells undergo ferroptosis.

### 3.5. SNX5 Promotes Ferroptosis in the PD Cell Model

**Figure 6:**
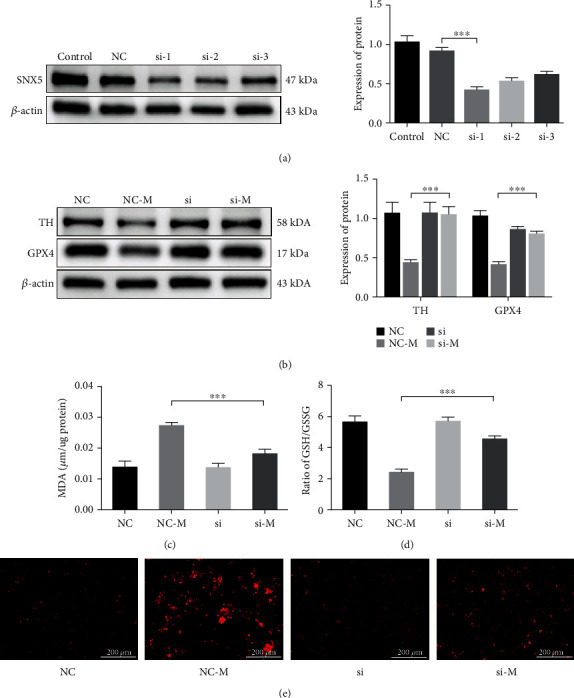
SNX5 promotes ferroptosis in the PD cell model. (a) Infection of PC-12 cells by conventional siRNA and screening the infected fragment with the best knockdown effect by Western blotting. PC-12 cells were infected with SNX5 transfected fragments before being treated with 40 *μ*M 6-OHDA for 24 h. The expression of TH and GPX4 was analyzed using Western blotting (b), and the effect of SNX5 knockdown on MDA (c), GSH/GSSG (d), and Fe^2+^(e) was detect by assay kit. Data were expressed as mean ± S.D.; three independent replicates of the experiment (^∗∗∗^*p* < 0.05).

To further validate the role of SNX5 in PD pathology and ferroptosis, we used siRNA interference fragments to knockdown the expression of SNX5 in the cell and then used 6-OHDA to stimulate the cell. Screening by experiment, the si-1 was the most efficient sequence for silencing (Figure 6(a)). After silencing SNX5 in PC-12 cells, administration of 6-OHDA stimulation, we found that knockdown the expression of SNX5 in the SI-M group can increase the expression of TH and GPX4 significantly compared to the NC-M group (Figure 6(b)). At the same time, the concentration of MDA decreased significantly, and the ratio of GSH/GSSG increased significantly in the SI-M group compared with the NC-M group (Figures 6(c) and 6(d)). In addition, the ferrous imaging results also confirmed that the SI-M group could result in reduced Fe^2+^ accumulation in the PD cell model (Figure 6(e)). These data further suggest that SNX5 plays a vital role in PD pathology and ferroptosis. In PD cell models, abnormal aggregation of SNX5 may affect the process of ferroptosis onset, leading to the accumulation of toxic lipid peroxides and reactive oxygen species (ROS) and finally causing ferroptosis of cells.

## 4. Discussion

This study determined that SNX5 is abnormally overexpressed in the PD model, and it can promote ferroptosis. In the previous work, the result shows that the expression of SNX5 increases significantly in the PD rat model by iTRAQ protein sequencing, and the high expression of SNX5 is driven by superenhancer [14]. However, the influence of SNX5 on PD and ferroptosis is not clear. SNX5, as the core protein of the endosomal sorting component retromer, significantly affects the morphology of the Golgi complex [20, 21]. The dimer formed by SNX5 is responsible for recruiting retromer into the endosome and is an essential protein involved in intracellular and intracellular material transport and signal transduction [16, 22]. In recent years, genomics animal and cell biology have shown that retromer-mediated protein transport defects in endosomal sorting are closely related to PD pathogenesis [15, 23, 24]. SNXs play a variety of roles in different aspects of endosomal transport, including the transport of endosomes to the trans-Golgi network, circulation to the cell surface, and endocytosis [25]. Retromer is also required for endosomatic circulation of iron transporter DMT1 [26], suggesting that endosomal sorting mediates part of the intracellular iron ion transport process.

In this study, the PD model was established by 6-OHDA, the PD pathology was enhanced, and the expression of SNX5 was increased significantly in the PD model. Besides, the ratio of GSH/GSSG and the expression of GPX4 in the model group were decreased, while the concentration of MDA was increased, suggesting that ferroptosis appeared in the PD model. Then, the ferroptosis inducer erastin and the ferroptosis inhibitor fer-1 were used to stimulate PC-12 cells. Erastin, as a classical ferroptosis inducer, can accelerate ROS accumulation in PC-12 cells by directly decreasing GSH activity to induce ferroptosis [27, 28]. In the experiment shown in Figure 5, the effects of 6-OHDA and erastin on ferroptosis were similar, and erastin can promote PD cell damage. Moreover, fer-1, as an inhibitor of lipid ROS production, can alleviate ferroptosis to a certain extent [29, 30]. When 6-OHDA and fer-1 are used in combination, fer-1 can inhibit the decrease of GPX4 and the increase of SNX5 in the PD cell model. Fer-1 could rescue PD cell model ferroptosis to a certain extent, suggesting that PD cells appear in ferroptosis and SNX5 is related to the appearance of ferroptosis. After the expression of SNX5 was inhibited by siRNA in PC-12 cells, the expressions of TH and GPX4 in the SI-M group were increased, the concentration of MDA was decreased, and the ratio of GSH/GSSG was increased significantly compared with the NC-M group. Compared with the SI group, the SI-M group showed no significant difference. In addition, the knockdown of SNX5 in PC-12 cells improved the intracellular accumulation of Fe^2+^. The results showed that the knockdown of SNX5 had a protective effect on PD and ferroptosis, indicating that SNX5 could promote ferroptosis in PD. However, the specific mechanism of how SNX5 regulates ferroptosis in PD is uncertain and needs to be further explored. Because previous studies have shown that SNX5 is one of the most important proteins in the endosome, which participates in the transport of multiple substances in the cell, including iron transport, SNX5 may influence the occurrence of ferroptosis by affecting iron transport.

## 5. Conclusion

In conclusion, this study demonstrated that abnormally high expression of SNX5 promotes ferroptosis in PD. The discovery of SNX5 also provides new insights into ferroptosis in PD, new diagnostic markers for PD diagnosis, and is expected to become a new therapeutic target for PD.

## Data Availability

The data and materials produced during the study can be obtained from the corresponding authors on reasonable request.
